# Grunt variation in the oyster toadfish *Opsanus tau*: effect of size and sex

**DOI:** 10.7717/peerj.1330

**Published:** 2015-10-15

**Authors:** Michael L. Fine, Tyler D. Waybright

**Affiliations:** 1 Department of Biology, Virginia Commonwealth University, Richmond, VA, United States; 2 Office of Student Assessment, Virginia Department of Education, Richmond, VA, United States

**Keywords:** Behavioral ontogeny, Bioacoustics, Sexual dimorphism, Sound production, Sonic muscles, Agonistic behavior

## Abstract

As in insects, frogs and birds, vocal activity in fishes tends to be more developed in males than in females, and sonic swimbladder muscles may be sexually dimorphic, i.e., either larger in males or present only in males. Male oyster toadfish *Opsanus tau* L produce a long duration, tonal boatwhistle advertisement call, and both sexes grunt, a short duration more pulsatile agonistic call. Sonic muscles are present in both sexes but larger in males. We tested the hypothesis that males would call more than females by inducing grunts in toadfish of various sizes held in a net and determined incidence of calling and developmental changes in grunt parameters. A small number of fish were recorded twice to examine call repeatability. Both sexes were equally likely to grunt, and grunt parameters (sound pressure level (SPL), individual range in SPL, number of grunts, and fundamental frequency) were similar in both sexes. SPL increased with fish size before leveling off in fish >200 g, and fundamental frequency and other parameters did not change with fish size. Number of grunts in a train, grunt duration and inter-grunt interval were highly variable in fish recorded twice suggesting that grunt parameters reflect internal motivation rather than different messages. Grunt production may explain the presence of well-developed sonic muscles in females and suggests that females have an active but unexplored vocal life.

## Introduction

As in insects, frogs and birds (Ryan, 1985; Gerhardt & Huber, 2002; Catchpole & Slater, 2008), vocal activity in fishes is typically more developed in males than in females (Amorim, Vasconcelos & Fonseca, 2015). One of the major mechanisms of sound production in fishes utilizes sonic muscles that drive the swimbladder to vibrate, and these muscles are often sexually dimorphic (Ladich & Fine, 2006; Fine & Parmentier, 2015; Ladich, 2015a). Sexual dimorphism in various fishes includes two separate morphological states. In some species, i.e., batrachoidids, gadids, ophidiids, osphronemids males have larger sonic muscles (Ladich, 2015a), but in others muscles may be present exclusively in males as in most species in the family Sciaenidae (Chao, 1978; Ono & Poss, 1982; Hill, Fine & Musick, 1987; Borie et al., 2014). However, some sciaenids, including Japanese croaker (Ueng, Huang & Mok, 2007), Atlantic croaker *Micropogonias chromis* (Hill, Fine & Musick, 1987), whitemouth croaker *M. funneri* (Tellechea et al., 2010a) and black drum *Pogonias chromis* (Tellechea et al., 2010b), have sonic muscles in both sexes. As in toadfish, sonic muscles and swimbladders are larger in male Atlantic croaker (Hill, Fine & Musick, 1987). To our knowledge the question of what would select for these two divergent patterns (size differences or absence) has not been formally addressed.

The oyster toadfish *Opsanus tau* L (Fine & Thorson, 2008) and other members of the family Batrachoididae have been used as model species for various aspects of acoustic communication (Bass & McKibben, 2003; Modesto & Canario, 2003a; Modesto & Canário, 2003b; Rome, 2006; Amorim & Vasconcelos, 2008; Rice & Bass, 2009; Rice, Land & Bass, 2011; Jordao, Fonseca & Amorim, 2012; Mosharo & Lobel, 2012; Vasconcelos et al., 2012; Bass, Chagnaud & Feng, 2015). Male *Opsanus tau* and *O. beta* produce a long-duration tonal courtship boatwhistle call, which functions in male-male competition and female attraction (Fish, 1954; Tavolga, 1958; Fine, 1978b; Edds-Walton, Mangiamele & Rome, 2002; Thorson & Fine, 2002b). Males will enter and call from shelters (Gray & Winn, 1961), which allows experimental manipulation by playbacks that demonstrate call rate, frequency and duration affect male boatwhistle production (Winn, 1967; Fish, 1972; Winn, 1972). Calls are produced by intrinsic sonic muscles that line the lateral walls of the swimbladder (Fine, Burns & Harris, 1990; Fine, Bernard & Harris, 1993; Barimo & Fine, 1998). Extremely rapid contraction of these muscles determines the fundamental frequency of the boatwhistle (Skoglund, 1961; Fine et al., 2001; Elemans, Mensinger & Rome, 2014). The sonic muscles and swimbladder occur in both sexes and grow for life (Fine, Burns & Harris, 1990). However, both are larger in males than in females, and males have more but smaller muscle fibers (Fine, Burns & Harris, 1990). Toadfish of both sexes produce a less-studied shorter-duration more pulsatile grunt call in agonistic situations (Fish, 1954; Tavolga, 1958; Gray & Winn, 1961). Maruska & Mensinger (2009) have provided the most detailed study of grunts utilizing an unseen semi-natural population of males and females in a long shallow outdoor tank; their paper provides sonagrams and oscillograms demonstrating extensive variation in toadfish grunts. They quantify occurrence and differentiate sound parameters of large numbers of single, doublet and trains of grunts that occurred spontaneously and net grunts from netted individuals. Their fish also produced boatwhistles suggesting fish were exhibiting normal courtship behavior even under captive conditions. Grunt parameters have also been measured from several identified male toadfish calling in the York River (Barimo & Fine, 1998), and grunts often precede boatwhistles in *Opsanus beta* from the Florida keys (Thorson & Fine, 2002b). Finally, Elemans, Mensinger & Rome (2014) recorded net grunts from fish with implanted emg electrodes demonstrating that each grunt is caused by a quick burst of several muscle contractions.

There has been little behavioral work on grunts. Territorial males grunt, particularly when guarding eggs, if tethered toadfish (both males and females) and even blue crabs were brought close to the male’s nest (Gray & Winn, 1961). Male toadfish have been found to grunt on top of boatwhistles and grunts of nearby males, an acoustic tag, suggesting a dominance display (Thorson & Fine, 2002a; Mensinger, 2014). Finally grunts have been evoked by electrical brain stimulation in *O. beta* (Demski & Gerald, 1972; Demski & Gerald, 1974) and *O. tau* (Fine, 1979; Fine & Perini, 1994). Grunts in *O. tau* were divided into knock and burst grunts (shorter and longer respectively) and indicate that the fish can vary the number of muscle contractions in a grunt pulse as well as the number of pulses and the timing between pulses. We are left with the impression that males would be more likely to grunt than females although this hypothesis has not been tested (Ladich, 2015a).

In this study we examined the effects of size and sex on grunt incidence and parameters by recording individual male and female oyster toadfish held in air (net grunts) to describe ontogenetic changes and potential sex differences. Effects of recording in air will be dealt with in the discussion. Because fundamental frequency (muscle contraction rate) is determined by pattern generators in the brain (Bass, Chagnaud & Feng, 2015), we hypothesized that unlike in most fishes (Ladich, 2015b), grunt fundamental frequency would not decrease with fish size. Further, due to increases in sonic muscle and swimbladder size in larger fish (Fine, Burns & Harris, 1990), we predicted that sound pressure level would increase with fish size. We find call parameters and incidence of calling are equivalent in males and females suggesting that females are more vocal than previously demonstrated and accounting for the presence of a well-developed sonic system in females.

## Materials and Methods

Oyster toadfish were collected in the York River, VA and kept in sea tables with running York-River water under ambient photoperiods at the Virginia Institute of Marine Science (VIMS). Other fish were transported to Virginia Commonwealth University (VCU) and kept in five 120 L tanks in half-strength artificial sea water (18‰) under a 14:10 LD cycle at 22 °C. Toadfish were weighed and then sexed using the presence of a cloaca (a second opening) between the anus and the urogenital papilla; the opening is present in females but not in males. A test of fish weighing between 113 and 786 g correctly determined the sex of 20 of 20 toadfish (10 males and 10 females) correctly as verified by gonadal inspection. Protocols were approved by the VCU Animal Care and Use Committee (IACUC no. AD20216).

VIMS fish, collected for immunology projects unrelated to the current study, were auditioned for the presence or absence of sound production at approximately monthly intervals from May 23 to September 1, 1989. Different individuals were used in the various trials. Grunt sounds were evoked by holding the fish in a small net, i.e., the net grunts of Maruska & Mensinger (2009). Some fish called immediately when netted, some required gentle prodding to call, and others remained silent.

VCU fish were recorded in air 20 cm from a Uher microphone and 2002 Report L tape recorder (Assmann Electronics, Bad Homburg, Germany) at 3.75 ips. Four of the smallest fish produced lower amplitude sounds and were recorded 5 cm from the microphone. Assuming spherical spreading (Urick, 1975; Fine & Lenhardt, 1983; Mann, 2006), we converted the amplitude of these sounds to the level expected at 20 cm by subtracting 15.6 dB or 20 log (20/5). Pumps were turned off in the aquarium room during recording. Fish were netted and brought to the microphone, which was adjacent to the tanks. The recording commenced when the fish started to call. Fish that made long and rapid trains of grunts were recorded until the fish stopped calling or grunts became infrequent. Likewise fish producing few grunts were provided time to potentially add additional grunts. For these reasons fish were not recorded for a standardized time.

Sounds were analyzed on a Kay 505 Sonagraph (Kay Elemetrics, Murray Hill, New Jersey, USA). A calibration tone produced using a function generator connected to a speaker was recorded through the microphone allowing amplitude determination of grunt sounds in dB re: 20 µPa (dB SPL). We also measured the period in ms of the most intense cycle in the waveform and used its reciprocal to calculate fundamental frequency. Since grunt calls can be highly variable (Maruska & Mensinger, 2009) and individuals varied in their incidence of grunting, we attempted to record 25 fish twice to determine the degree of call fixity, i.e., do individual toadfish have a signature grunt. Seven of these fish called twice. For these fish we measured temporal parameters including number of grunts, grunt duration, and inter-grunt interval, the time between grunt pulses as measured from the call waveform.

For calling incidence, we set up contingency tables of calling and silent males and females and used Fisher’s exact probability test to determine sexual differences (GraphPad software, San Diego, California, USA). For call parameters, means for each fish were plotted against fish weight to determine developmental changes and sex differences. Data were fit with linear regressions except for SPL which was fit with a one phase association equation. Regressions for males and females were compared with analysis of covariance using fish weight as the covariate. For SPL comparisons, data were linearized with a log–log transform before comparison. If regression slopes were not significantly different from zero in both sexes, we used a *t*-test to compare parameters by sex. Male and female regressions were combined if sex differences were not significant. Temporal data for fish recorded twice were compared by regressing means from the first recording against means from the second.

## Results

### Grunt incidence in males and females

**Figure 1:**
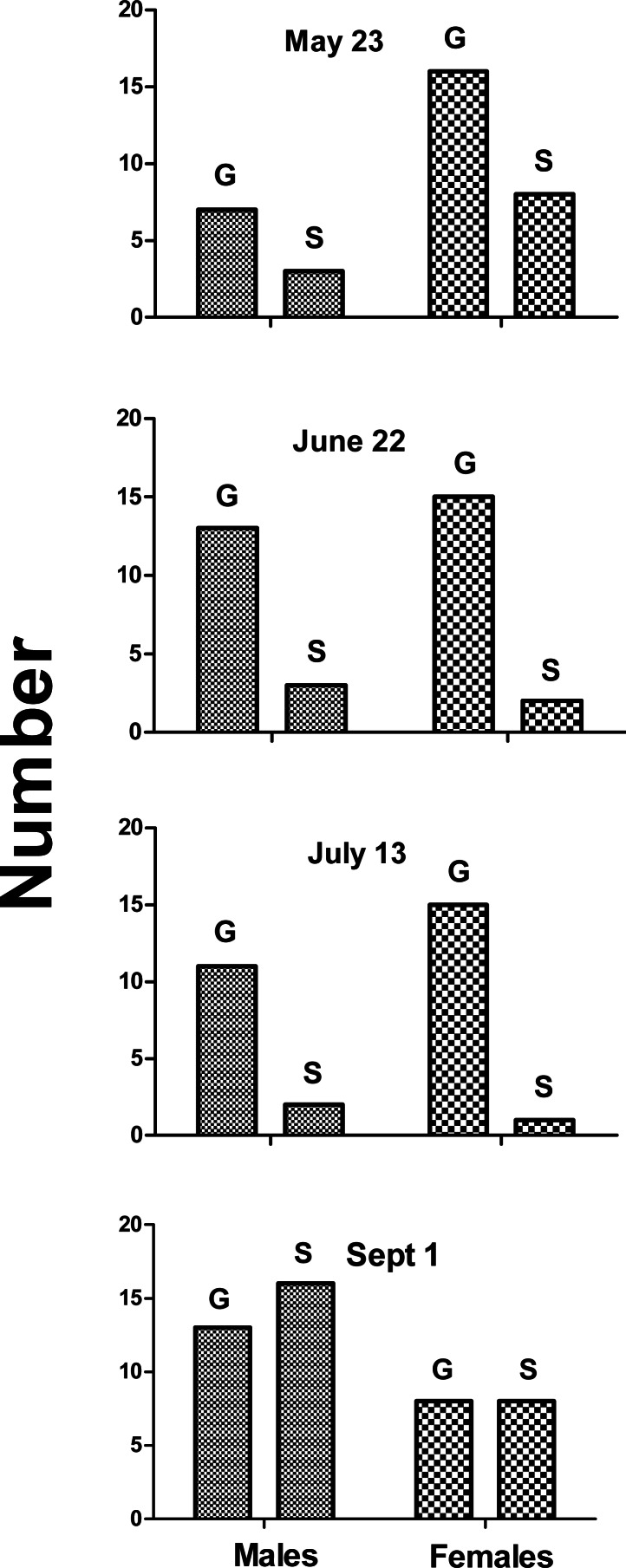
Number of males and females that grunted (G) or were silent (S) on indicated dates. Males and females were equally likely to grunt on all four dates (Fisher exact probability test), and incidence of grunting decreased in September.

Over four recording sessions, we auditioned 128 different toadfish (59 males and 69 females) ranging in size from 15 to 835 g. Fisher’s Exact Probability test indicated no significant difference between the incidence of grunt production between males and females on any of the trial dates (Fig. 1). There was a decreased incidence in both sexes on Sept 1. Respectively 70% and 67% of males and females grunted on May 23 (*P* = 1.0), 82% and 88% on June 22 (*P* = 0.66), 81% and 94% on July 13 (*P* = 0.57), and 45% and 50% on Sept. 1 (*P* = 0.77). Thus males and females were equally likely to produce grunts when held.

### Effects of size and sex on grunt parameters

**Figure 2:**
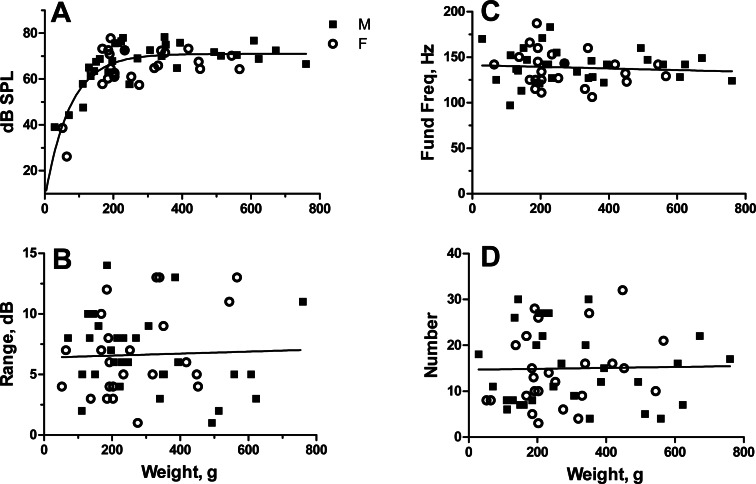
Relationship of sound pressure level in dB re: 20 µPa (A), range in dB (B), fundamental frequency (C) and number of grunts (D) to fish weight for male and female oyster toadfish. Points represent the mean value for each individual except for number of grunts, which is the actual number. There were no sexual differences, and the regression line represents pooled data for males and females.

Male toadfish ranged between 29 and 760 g and females between 52 and 567 g. The largest four fish were males, which grow to larger sizes than females (Radtke, Fine & Bell, 1985). SPL increased nonlinearly from 39 to 78 dB SPL in males and 26 to 78 dB in females. Increase was rapid in both sexes to about 200 g and then leveled off (Fig. 2A). Analysis of covariance on regressions linearized with log–log transforms indicated no difference in SPL between males and females (Slopes: *F*_1,52_ = 2.59, *P* = 0.12; Intercepts: *F*_1,53_ = 1.65, *P* = 0.20). Data points for males and females co-scattered, and much of the difference between slopes came from one female with the lowest amplitude. Male and female data were combined and fit with a one-phase exponential equation ((*y* = 7.084 + 63.936 (1 − e^−0.01378*x*^)) with an *r*^2^ of 0.64 (Fig. 2A).

SPLs for individuals ranged from 1 to 14 dB with a mean of 6.6 ± 3.3 dB (SD) in males and 1–14 dB (mean 6.6 ± 3.6 dB) in females. Since range did not change with fish size (*r*^2^ = 0.002, *P* = 0.78) (Fig. 2B), mean range for males and females was compared with a *t* test indicating no sexual difference (*t*_52_ = 0.021, *P* = 0.98).

Fundamental frequency ranged from 97 to 183 Hz (mean 139.8 ± 18.2 Hz) in males and 106 to 187 Hz (mean 137.3 ± 19.6) in females and did not vary with fish size (*r*^2^ = 0.007, *P* = 0.56) (Fig. 2C). Fundamental frequency was similar between males and females (*t*_52_ = 0.49, *P* = 0.62).

Number of grunt pulses varied from 4 to 30 (mean 15.5 ± 8.5) in males and 3 to 32 (mean 14.4 ± 8.0) in females and did not change with fish size (*r*^2^ = 0.0004, *P* = 0.88) (Fig. 2D). Number of grunt pulses per fish was again similar between males and females (*t*_52_ = 0.49, *P* = 0.63).

### Temporal properties and repeatability

**Figure 3:**
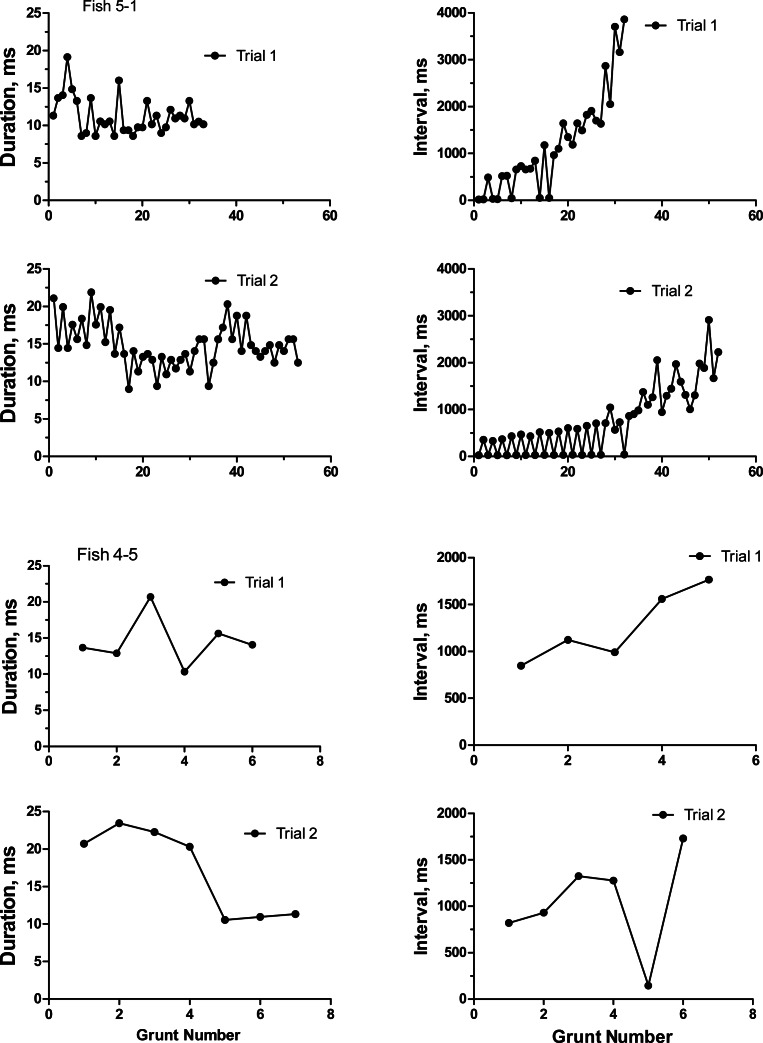
Duration and inter-grunt interval in milliseconds during the course of a grunt train for two oyster toadfish recorded on two occasions 6 days apart.

**Figure 4:**
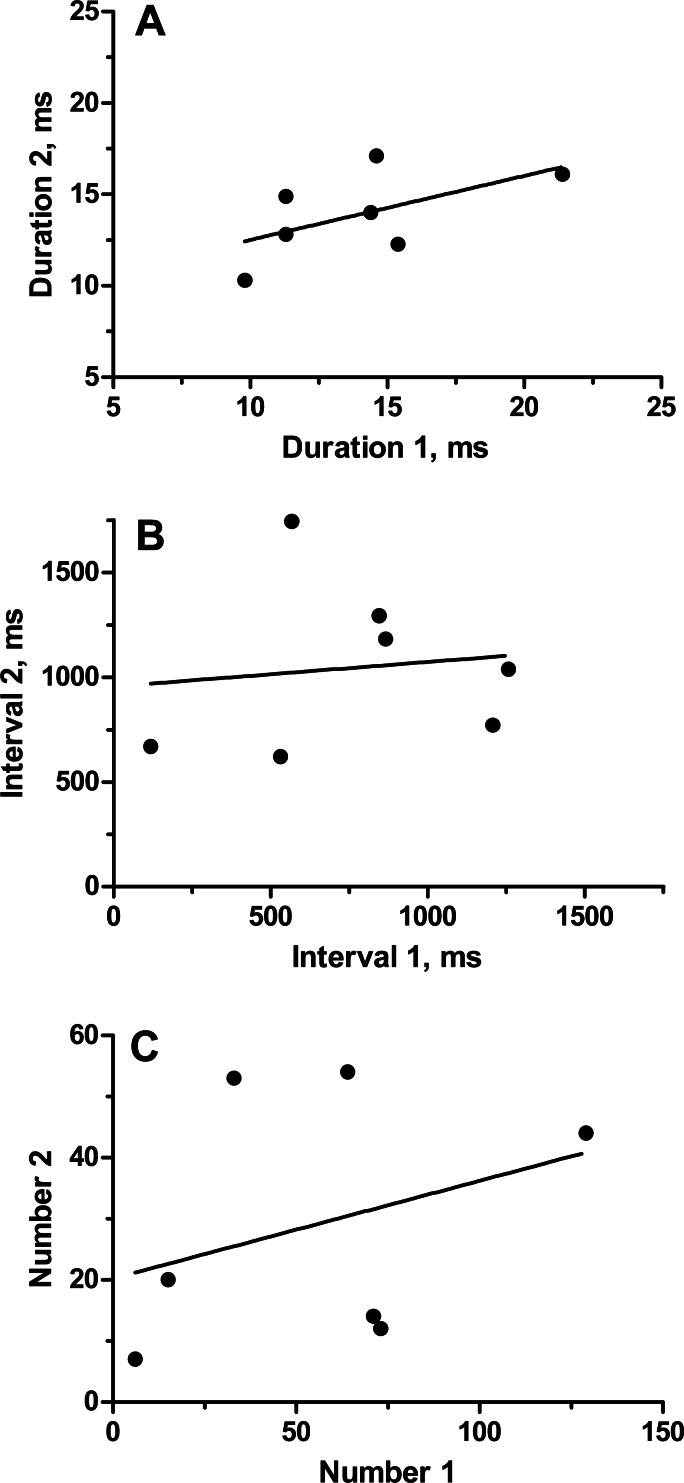
Relationship of parameters (average grunt duration, inter-grunt interval and number of grunts) for seven individual oyster toadfish recorded on two occasions.

To categorize the variability and repeatability in grunts produced by individual toadfish, we recorded seven fish twice (August 19 and 24, 1989). Grunts were emitted in trains of pulses that varied extensively in number of grunts, inter-grunt interval and to a lesser extent in grunt duration (Table 1). Number of grunts across all fish varied from 6 to 129, and most grunts were rather short with a range in means from 10.3 to 21.3 ms. Inter-grunt intervals within trains were highly variable and tended to be bimodal with bursts of pulses wedged in between longer pauses coupled with a general trend for longer pauses toward the end of a grunt train (hence multimodal) (Fig. 3). Individual, pairs and burst grunts occurred. Means or medians do not adequately describe the intervals, which for fish 5-1 ranged between 18 ms to almost 4 s (Fig. 3). Two examples were chosen to illustrate the duration and inter-grunt interval of each grunt in a train recorded on both days (Fig. 3). These examples illustrate the range in variability among fish and suggest a degree of similarity between recording dates. Similarity was not evident in every individual (Table 1 and Fig. 4). Comparisons between pairs of recordings for individuals indicated a lack of correlation between sessions (Fig. 4): for duration (*r*^2^ = 0.33, *P* = 0.17), inter-grunt interval (*r*^2^ = 0.014, *P* = 0.80), number of grunts (*r*^2^ = 0.11, *P* = 0.47). Therefore even when there appear to be trends in qualitative pattern, numbers of grunts and quantitative measurements of their patterning are quite different. The number of grunts from individuals in the two recordings ranged from six and seven grunts (similar), 33 and 55 grunts (somewhat similar), to 73 to 12 (quite dissimilar). Although we suspect some toadfish may exhibit tendencies in the patterning of their grunts, overall the calls are extremely variable and did not demonstrate individual signatures.

**Table 1:** Mean ± SD, minimum and maximum value for grunt duration, inter-grunt interval and number of grunts for seven individual oyster toadfish recorded on two separate days (a and b).

	Grunt duration, ms			Inter-grunt interval, ms			
Fish	$\bar {x}\pm \mathrm{SD}$	Minimum	Maximum	$\bar {x}\pm \mathrm{SD}$	Minimum	Maximum	*N*
3-1 a	21.3 ± 6.4	12.1	54.0	840 ± 1,143	19.9	7,925	64
b	16.1 ± 4.0	11.7	26.6	1,182 ± 561	41.4	3,625	54
3-2 a	9.8 ± 3.1	4.7	18.0	118 ± 384	7.8	2,294	129
b	10.3 ± 2.3	5.0	17.6	669 ± 2,935	12.5	18,105	44
4-4 a	11.3 ± 3.7	4.7	16.0	845 ± 920	19.2	3,100	15
b	12.8 ± 4.0	7.8	21.9	1,293 ± 1,183	23.8	4,200	20
4-5 a	14.6 ± 3.1	10.6	20.7	1,257 ± 349	847	1,766	6
b	17.1 ± 5.4	10.6	23.4	1,038 ± 496	145	1,731	7
5-1 a	11.3 ± 2.4	8.6	19.1	1,207 ± 1,042	18.0	3,862	33
b	14.9 ± 2.9	9.0	21.9	771 ± 702	25	2,912	53
5-3 a	15.4 ± 1.3	12.1	18.8	531 ± 1,190	17.6	9,455	73
b	12.3 ± 0.6	10.5	12.9	620 ± 430	24.6	1,600	12
5-5 a	14.4 ± 3.4	7.8	23.8	567 ± 1,007	13.7	4,238	71
b	14.0 ± 5.9	7.0	28.1	1,743 ± 3,211	19.1	12,400	14

## Discussion

Toadfish live in murky water in Atlantic estuaries (Able & Fahay, 1998), and besides boatwhistles little is known about behavioral and acoustical interactions under natural conditions. We recorded individuals in air, which has the advantage of subjecting individuals to an equivalent stimulus under reasonable if abnormal acoustic conditions. Although underwater recordings would be preferable, tanks have complex acoustic fields that can alter signal frequency spectra due to resonance and decrease sound levels because of out-of-phase reflections from tank boundaries (Akamatsu et al., 2002; Parmentier et al., 2014). It would be difficult to record large numbers of toadfish underwater in close to free-field conditions. Three studies have examined calls from individuals of the same species recorded in air and underwater: two on pectoral stridulation sounds in catfishes (Knight & Ladich, 2014; Ghahramani, Mohajer & Fine, 2014) and one on swimbladder sounds in Atlantic croaker (Fine, Schrinel & Cameron, 2004). Croaker sounds therefore provide the most apt comparisons. Croakers were recorded in a shallow large boat harbor, which minimized but did not remove all acoustic complications from potential reflections. The peak frequency of croaker sounds in both media was identical because it is determined by timing of sonic muscle contractions as in the toadfish (Skoglund, 1961; Fine et al., 2001; Elemans, Mensinger & Rome, 2014) and not bladder resonance (Fine, 2012; Fine & Parmentier, 2015). Underwater croaker sounds damped more slowly (the waveform contained an extra cycle) and were more sharply tuned (higher Q) than in air, and it is likely therefore that grunts in this study would be several milliseconds longer if recorded underwater.

Fish sounds have been divided into fixed and variable interval calls (Winn, 1964), and the variability in toadfish grunts is striking. Grunts recorded at Woods Hole, Massachusetts were also variable and described as single, doublet and trains of grunts (Maruska & Mensinger, 2009). All three of these grunt types were emitted in this study, suggesting as in birds (Brown, 1975) different calls can occur in the same behavioral context. Long trains of variable-interval grunts with intervals increasing over time are reminiscent of following grunts evoked after the cessation of electrical brain stimulation (Fine, 1978a). Net grunts recorded by Maruska & Mensinger had an average duration of 178 ms, approximately ten fold longer than ones recorded in this study. Part of this difference could be due to geographical variation (Fine, 1978a; Fine, 1978b) between populations in Virginia and Woods Hole, Massachusetts, the northern limit of the toadfish’s geographical range. More likely, we suggest a difference in the fish’s central state since their outdoor tank included territorial males who boatwhistled during the mating season whereas fish in this study were held in smaller indoor tanks. The waveform of many of the Woods Hole grunts was somewhat boatwhistle-like and made by multiple muscle contractions in rapid succession whereas individual grunts in this study were caused by a single or small number of contractions (Fine et al., 2001; Elemans, Mensinger & Rome, 2014). Grunts recorded from identified males that were boatwhistling in the York River, VA fish varied from 48 to 147 ms in duration (Barimo & Fine, 1998), supporting the hypothesis that the grunts reflect different internal states of the fish under courtship and distress situations. Similar to the Woods Hole study (Maruska & Mensinger, 2009), the incidence of grunts decreased in September. The active part of the mating season ends in July in Chesapeake Bay estuaries, after which toadfish reduce calling (Fine, 1978b).

### Behavior ontogeny

With the exception of SPL, grunt parameters were extremely variable but stable in fish from 29 to 760 g, equivalent to >100 to over 300 mm total length (Fine, 1975) and at least two to over 10 year-old individuals (Radtke, Fine & Bell, 1985). Although no juveniles were recorded in this study, we have heard grunts from fish <60 mm total length suggesting a lack of major changes in calls over most of the lifespan of the fish. Extreme variability even in fish recorded twice suggests that call variability may reflect the internal state at the moment rather than indicate different messages.

Although fish sounds typically increase in amplitude and decrease in frequency with fish size (Myrberg, Ha & Shamblott, 1993; Henglmüller & Ladich, 1999; Connaughton, Taylor & Fine, 2000; Wysocki & Ladich, 2001; Ladich, 2007; Lechner, Wysocki & Ladich, 2010; Tellechea et al., 2010a; Tellechea et al., 2010b), toadfish grunts differ in some ways from this pattern. SPL increased with size to 200 g and then unexpectedly stabilized. Sound amplitude is related to volume velocity (Bradbury & Vehrencamp, 1998), the product of speaker surface area and velocity of movement. Since swimbladder size increases continuously and dominant frequency does not change, sound amplitude should continue to increase as demonstrated with electrically-stimulated twitch sounds (Fine et al., 2001). However, that study increased stimulation voltage until maximal sound amplitude stabilized to ensure complete muscle fiber recruitment. In the current study dB ranges could be as much as 15 dB indicating that fish can control the amplitude of their sounds and potentially obscure a size effect on amplitude. It could also account for the lack of difference in SPL of male and female grunts. SPL increased continuously with fish size in the Portuguese toadfish *Halobatrachus didactylus* (Vasconcelos & Ladich, 2008) recorded in a shallow (12 cm) tub (30 × 45 cm). However, they plotted toadfish length data on a log scale suggesting that SPL in fact levels off in larger fish. Pulse period decreased with fish size suggesting longer sonic muscle twitch times in this species, which would at least partially explain a leveling off of SPL in amplitude in *Halobatrachus*. We are unsure why SPL levels off in the current study.

The absence of size effects on fundamental frequency was predicted because this variable is controlled by central pattern generators (Bass, Chagnaud & Feng, 2015) and twitch timing of the sonic muscles (Fine et al., 2001; Millot, Vandewalle & Parmentier, 2011) rather than resonant frequency of the swimbladder (Fine, 2012; Fine & Parmentier, 2015). Lower frequency sounds in larger fishes has historically been interpreted to result from lower resonant frequencies of larger swimbladders (Harris, 1964; Bergeijk, 1964). More recent work with swimbladder sounds suggests that apparently resonant sounds, ones that damp slowly, result from continuous vibration of a bone or tendon that continues to drive the swimbladder into vibration as in squirrelfish, glaucosomatids and carapids (Fine & Parmentier, 2015). Other cases in which swimbladders are driven directly by muscle twitches as in weakfish (Connaughton, Taylor & Fine, 2000) and *Halobatrachus* (Vasconcelos & Ladich, 2008) have more modest slopes when plotted against fish size because larger muscles take longer to complete a twitch and therefore produce longer pulses (Connaughton, Fine & Taylor, 2002; Parmentier & Fine, in press). We believe that the current study provides the first explicit demonstration that swimbladder size does not affect sound frequency although boatwhistle choruses recorded in the field (Fine, 1978b) contained fundamental frequencies that varied over as little as 10 Hz, therefore pointing to the same conclusion.

An ontogenetic study of hearing and sound production in *Halobatrachus* indicated that SPL increased, but sound duration, number of sounds and dominant frequency decreased with fish standard length (3–32 cm SL) (Vasconcelos & Ladich, 2008). Many of their smallest fish failed to grunt. The auditory brainstem response indicated that hearing was best below 300 Hz in fish of all sizes but that the smallest juveniles had lowest sensitivities (higher thresholds) at low (100 Hz) and high (800 and 1,000 Hz) frequencies. The authors suggest that acoustic communication might be absent in young juveniles. Auditory sensitivity has been determined in the oyster toadfish (Fish & Offutt, 1972; Yan et al., 2000) but only in adults, which also hear best below 300 Hz but appear to be less sensitive than *Halobatrachus*.

### Male and female grunts

Incidence of grunting as well as number of grunts, grunt duration, inter-grunt interval and sound pressure level were similar in males and females. It is clear that both sexes possess a similar grunt repertoire under these conditions. Based on swimbladder and sonic muscle size and general assumptions, we would not have predicted these findings. Considering the possibilities of either smaller or no muscles in females of various species, this study suggests that female oyster toadfish participate in a vocal world and may account for their well-developed albeit smaller sonic system.

What would select for loss of sonic muscles in many female sciaenid species has not been formally considered. Assuming that both sexes in an ancestral sciaenid (Lo et al., 2015) had sonic muscles originally, the simplest hypothesis would be that females lost muscles in species in which sound function was restricted to male advertisement calls, and females had no role in sound production. However, male weakfish *Cynoscion regalis* and silver croaker *Plagioscion squamisissimus* produce advertisement calls (Connaughton & Taylor, 1996; Borie et al., 2014) but also disturbance calls when held (Connaughton, Fine & Taylor, 2002; Borie et al., 2014). Only males have sonic muscles in these species (Ono & Poss, 1982; Hill, Fine & Musick, 1987). Disturbance calls likely function in predator–prey interactions (Bosher, Newton & Fine, 2006) and should be equally useful for both sexes. Accordingly, the evolution of sexual dimorphism in fish sonic mechanisms and what would select for mute females requires further exploration.

## Conclusions

Even though male toadfish produce a courtship boatwhistle and possess larger sonic swimbladder muscles than females, both sexes are equally likely to grunt under distress conditions. Grunts are extremely variable, and this variation is similar in both sexes. The lack of a size effect on fundamental frequency supports the notion that sounds are produced as a forced rather than a resonant response. We suggest that grunt variation reflects internal state of the caller rather than different messages and that calling in females explains the presence of sonic muscles, which are lost in some species of sonic fishes.

## Supplemental Information

10.7717/peerj.1330/supp-1 Supplemental Information 1 Toadfish grunt raw data
